# Genome-wide analysis of *Claviceps paspali*: insights into the secretome of the main species causing ergot disease in *Paspalum* spp

**DOI:** 10.1186/s12864-021-08077-0

**Published:** 2021-10-26

**Authors:** H. Oberti, G. Spangenberg, N. Cogan, R. Reyno, M. Feijoo, S. Murchio, M. Dalla-Rizza

**Affiliations:** 1 Instituto Nacional de Investigación Agropecuaria (INIA). Unidad de Biotecnología. Estación Experimental INIA Las Brujas, Ruta 48 km, 10 Canelones, Uruguay; 2 Agriculture Victoria, AgriBio, Centre for AgriBioscience, 5 Ring Road, Bundoora, VIC 3083 Australia; 3 School of Applied Systems Biology, La Trobe University, 5 Ring Road, Bundoora, VIC 3083 Australia; 4 Instituto Nacional de Investigación Agropecuaria (INIA). Programa Pasturas y Forrajes. Estación Experimental INIA Tacuarembó, Ruta 5 km, 386 Tacuarembó, Uruguay; 5 Centro Universitario Regional del Este (CURE), Polo de Desarrollo Universitario: Patogenicidad, toxicidad y genética en los ecosistemas pastoriles de la región Este de Uruguay, Ruta 8 km, 281 Treinta y Tres, Uruguay

**Keywords:** Biotroph, Comparative genomics, Effectors, Pathogenicity factors, In silico prediction

## Abstract

**Background:**

The phytopatogen *Claviceps paspali* is the causal agent of Ergot disease in *Paspalum* spp., which includes highly productive forage grasses such as *P. dilatatum*. This disease impacts dairy and beef production by affecting seed quality and producing mycotoxins that can affect performance in feeding animals. The molecular basis of pathogenicity of *C. paspali* remains unknown, which makes it more difficult to find solutions for this problem. Secreted proteins are related to fungi virulence and can manipulate plant immunity acting on different subcellular localizations. Therefore, identifying and characterizing secreted proteins in phytopathogenic fungi will provide a better understanding of how they overcome host defense and cause disease. The aim of this work is to analyze the whole genome sequences of three *C. paspali* isolates to obtain a comparative genome characterization based on possible secreted proteins and pathogenicity factors present in their genome. In planta RNA-seq analysis at an early stage of the interaction of *C. paspali* with *P. dilatatum* stigmas was also conducted in order to determine possible secreted proteins expressed in the infection process.

**Results:**

*C. paspali* isolates had compact genomes and secretome which accounted for 4.6–4.9% of the predicted proteomes. More than 50% of the predicted secretome had no homology to known proteins. RNA-Seq revealed that three protein-coding genes predicted as secreted have mayor expression changes during 1 dpi vs 4 dpi. Also, three of the first 10 highly expressed genes in both time points were predicted as effector-like. CAZyme-like proteins were found in the predicted secretome and the most abundant family could be associated to pectine degradation. Based on this, pectine could be a main component affected by the cell wall degrading enzymes of *C. paspali*.

**Conclusions:**

Based on predictions from DNA sequence and RNA-seq, unique probable secreted proteins and probable pathogenicity factors were identified in *C. paspali* isolates. This information opens new avenues in the study of the biology of this fungus and how it modulates the interaction with its host. Knowledge of the diversity of the secretome and putative pathogenicity genes should facilitate future research in disease management of *Claviceps* spp.

**Supplementary Information:**

The online version contains supplementary material available at 10.1186/s12864-021-08077-0.

## Background

About 59 species of *Claviceps* cause the ubiquitous ovary restricted ergot disease, [1] affecting over 600 monocot plants including forage and the main cereals. *Claviceps paspali* is the main source of ergot disease in *Paspalum* spp. [2–4]. This fungus behaves as a true biotroph during its infection *in planta*, although it can be easily grown in axenic culture [5]. The genus *Paspalum* includes important forage grasses. Some of them, such as *P. dilatatum,* has all the desired characteristics for adaptation to a climate change scenario, such as being highly productive, drought and grazing tolerant, and persistent [6–9]. Infection with Ergot results in quantitative/qualitative loses of seed production by seed replacement with the sclerotia of the fungus [10], and the production of tremorgenic mycotoxins like paspalitrem A and B [1, 10], which is toxic to feeding animals [1, 11, 12]. Susceptibility to Ergot disease has prevented some cultivars of the genus from reaching their maximum potential [13, 14], and its importance becomes crucial where beef and dairy production are based on natural pastures, like Uruguay and Argentina. The agronomic impact in these grasses are largely because of the lack of a reported effective genetic resistance in the genus while fungicides can only marginally control the disease [15, 16].

There are various mechanisms of pathogen virulence and a wide range of plant immune responses [17]. Colonization by biotrophic fungus like *C. paspali* with compatible interaction could be a complex cross-talk between pathogen-host. This cross-talk involves hundreds of secreted fungal molecules, including enzymes that inactivate antimicrobial compounds produced by the host [18, 19], plant cell wall-degrading enzymes (PCWDEs) [20], transporters for acquiring nutrients, effector proteins and small molecules [21], among others. The main function of extracellular proteins is to interact with the environment of a fungus which is of paramount importance for the interaction of a pathogen with its host [21–23]. Although plant pathogenic fungi can secrete a large number of proteins, only a small proportion of these have been characterized as pathogenicity factors [24]. Some of these are effectors expressed *in planta* that suppress plant defense response by modulating plant cellular metabolic pathways and signaling cascades, and interfering with recognition machinery like cell wall receptors [25–28]. Another reported function for effectors is to modulate plant physiology to accommodate fungal invaders and provide them with nutrients [29, 30]. To recognize pathogens, hosts have evolved the receptor protein immunity system that identifies effectors and pathogen/microbe associated molecular patterns (PAMPs/MAMPs). Infected plants can then initiate first-line innate PAMP-triggered immunity (PTI) or second-line effector-triggered immunity (ETI). PTI is initially triggered by PAMP (chitin is the most common in fungi) on the cell surface [18, 31]. Effectors like LysM containing domains Slp1 of *Magnaporthe oryzae* [32] or Ecp6 of *Cladosporium fulvum* [33], could suppress PTI from interfering with the recognition mechanism. ETI involves the recognition of the secreted effector by resistance-mediating receptor proteins (R-proteins), which trigger a strong defense response [34]. This mechanism leads progressively to the formation of pathogenic races with differences in the effector repertory [35, 36]. These differences are due to loss or modifications of effectors that can avoid detection through the corresponding arsenal of R-genes in the host plant. Meanwhile, the modification of the effector repertory places a selection pressure on the host for the formation of new R-gene variants [37].

Fungi with highly plastic genomes provide genetic diversity that not only is important in host or environmental adaptations, but also in contributing to divergence and speciation, like happened in *Claviceps* genus with *C. purpurea* [38]. Single species genomics and comparative genomics are important tools to study fungi adaptation to occupy environmental niches through the acquisition, loss, or diversification of protein families like effectors [39]. The value of comparative genomics to study the evolution of pathogenesis and the discovery of novel virulence determinants has been proved in several plant pathogen fungi genus like *Fusarium* [40, 41]*, Botrytis* [42, 43] and *Rhizoctonia* [44]*,* among others. But, only a limited number of studies have been conducted to identify secreted protein genes and possible pathogenicity factors in *Claviceps* genus, all of them centered in *C. purpurea.* Lo Presti et al. [18] performed the first genome-wide in silico secretome analysis of the *Claviceps* species together with several other fungus with different lifestyles. These authors discovered a low proportion of PCWDEs and a high proportion of not known functional secreted proteins in all the *Claviceps* analyzed, as expected in the biotrophic lifestyle. The presence of several genes like *cppg1/cppg2* [45]*, cptf1* [46]*, cpmk1* and *cpmk2* [47]*,* involved in *C. purpurea* pathogenicity were detected, but none of them have been reported in *C. paspali*. Despite the availability of sequenced *Claviceps* spp. genomes and protein data, no expression data had been reported for *C. paspali*. There is no knowledge of the genes involved in causing disease, how it avoids necrosis or hypersensitive response in its host, or how it maintains its host’s cell viability to obtain nutrients from living tissue [23, 48–50].

Specialized *Claviceps* genes involved in host–pathogen interactions such as host-specific effectors, elicitors, or Avirulence genes are undefined. Understanding *C. paspali* evolution is critical and valuable not only for predicting and monitoring the population changes but also for developing cultivars with durable resistance [51]. The use of functional genomics that use well-characterized effectors for the detection of R-genes in plant germplasm for the production of cultivars with long-lasting resistance is becoming increasingly common [35–37], and this could be used in *C. paspali* resistance.

Here, we used in silico prediction to identify possible secreted proteins and pathogenicity factor-like proteins present in the genomes of three isolates of *C. paspali* belonging to different lineages [52, 53] within the section *Paspalorum* [1]. Then, we used *in planta RNA-seq* during the early stages of infection of a *P. dilatatum* susceptible cultivar by *C. paspali* to pinpoint the expressed secretome. Since the related pathogens might differ in their secretome mainly by gene gain/loss or by rapid evolution of shared pathogenicity factors, we compared the full set of predicted secreted proteins of *C. paspali* with two other *Claviceps* species (*C. purpurea* and *C. fusiformis*) that are closely related but have completely different plant hosts. The analysis of secreted and pathogenicity factor-like proteins encoded in the genome should help to understand how the fungus manages to avoid plant defense response leading to pathogen perpetuation without killing the host (47). In turn, this would be useful for the identification of resistant counterparts in *Paspalum* spp. and for designing efficient strategies against this poorly characterized pathogen.

## Results

### Genome assembly and annotation of ILB432 and ILB388 genome

*C. paspali* ILB388 isolate from *P. plicatulum* belongs to a separate lineage from the previously sequenced *C. paspali* isolates [53, 54]. The newly sequenced ILB388 has a larger genome than previously reported for *C. paspali* genome and slight difference in GC content and repeated sequences (Table 1). Based on a Chi-square test, differences between GC content was not significant (*p* < .01) between isolates. Null hypothesis was mean GC content of isolates, and chi-square statistic was 0.0267 and *p*-value .986741. A BUSCO assembly completeness analysis on 3156 orthologous genes for Ascomycota recovered 98.7, 98.4 and 96.9% single copy orthologues in genome assembly for ILB432, ILB388 and RRC-1481 respectively.

**Table 1:** Comparison of genome assembly stats of *Claviceps paspali* isolates

Category	ILB388	ILB432	RRC-1481
Estimated genome size (Mb)	29.2	28.9	28.9
GC content (%)	49.4	47.8	48.0
No. of contigs	885	701	2304
N_50_ of contigs	89,397	79,562	26,898
No. of scaffolds (> 200 bp)	586	352	n/s
Total length of scaffolds (bp)	29,270,071	28,980,376	n/s
N_50_ of scaffolds (bp)	196,155	146,886	n/s
Longest scaffolds (bp)	734,927	616,281	n/s
No. N’s per 100 kb	21.0	23.0	0.8
BUSCO analysis	98.4(S),0.0(D),0.5(F),1.1(M)	98.7%(S),0.0%(D),0.1%(F),1.2%(M)	96.9(S),0.0(D),1.2(F),1.9(M)
Repeat sequences (%)	23.00	22.34	22.39

S=Complete single copy; D=Complete Duplicated; F=Fragmented; M = Missing; n/s = non-scaffold assembly level.

For the integrated de novo gene prediction of the three *C. paspali* isolates, the FunGAP pipeline was used with default parameters, in a combination of ab initio predictions, assembled RNA-seq transcripts obtained from four plant-pathogen mixed libraries **(**Additional file S1), and proteins from three related *Clavicipitaceae.* An annotation summary is displayed in Table 2**.** A total of 8012, 8243 and 8122 high-confidence protein-coding genes were predicted for ILB388, ILB432 and RRC-1481 respectively.

**Table 2:** Summary of genome annotation for *Claviceps. paspali* isolates

	ILB432	ILB388	RRC-1481
Total protein-coding genes	8243	8012	8122
Transcript length (avg / med)	1593.6 / 1344.0	1633.6 / 1389.0	1587.3 / 1291.0
CDS length (avg / med)	1395.9 / 1155.0	1430.1 / 1186.5	1384.1 / 1119.0
Protein length (avg / med)	465.3 / 385.0	476.7 / 395.5	461.4 / 373.0
Exon length (avg / med)	532.5 / 262.0	539.4 / 271.0	557.1 / 291.0
Intron length (avg / med)	121.9 / 96.0	123.3 / 97.0	136.8 / 98.0
Spliced genes	6141 (74.5%)	5924 (73.94%)	5584 (68.75%)
Gene density (genes/Mb)	284.43	273.73	280.32
Number of introns	13,367	13,229	12,057
Number of introns per gene (med)	2.0	2.0	2.0
Number of exons	21,610	21,241	20,179
Number of exons per gene (med)	2.0	2.0	2.0

### Prediction of encoded candidate secretome in *Claviceps paspali* isolates

We identified a comprehensive set of probable secreted proteins (PSP) in *C. paspali* isolates causing Ergot in *Paspalum* spp. Combining prediction programs increase specificity but decrease sensitivity, meaning that false positives results are excluded, and true positives are lost [55]. Due to this fact, we intended to make a more stringent search to report the most probable PSP in the *C. paspali* isolates. Proteins predicted to have a signal peptide, with no GPI anchor and TM domain and no ER retention signal with a predicted subcellular localization as extracellular were classified as secreted. From the full set of proteins of *C. paspali* isolate RCC-1481, 437 of these were considered as PSP, 371 were predicted in isolate ILB388, and 407 in isolate ILB432. The three isolates ILB388, ILB432 and RRC-1481 presented a similar small proportion of PSP ranging from 4.6–4.9% of the whole predicted proteome (Table 2).

### Comparison of functional annotations between encoded predicted secretome candidates in *Claviceps paspali* isolates

The predicted secreted proteins were further functional annotated based on comparisons with the Nr NCBI database. Among the 1212 total PSP a BLAST hit was obtained in 248 in ILB388, 269 in ILB432 and 234 in RRC-1481. Functional annotation of the three isolates showed that and only 27.5% could be annotated with Gene Ontology (GO) terms (121 in ILB388, 115 in ILB432 and 117 in RRC-1481). Also, only 31 in ILB432, 25 in ILB388 and 32 in RRC-1481 sequences could be mapped to the KEGG pathway database.

Statistical comparisons between functions predicted in the three isolates were performed using Fisher’s exact test (*p* < 0.05) (Additional file S2). Hydrolase activity (GO:0016787) was the most common molecular function of the predicted secretome for each *C. paspali* isolates. RRC-1481 has the highest number of proteins with hydrolase activity (77), while ILB432 has the lowest number (18). The annotations of the PSP in the GO Biological Process domain were mainly related to carbohydrate metabolic processes (GO:0005976, GO:0000272, GO:0044262), where RRC-1481 had the highest number of proteins (35) and ILB388 and ILB432 the lowest (17). Annotations in the GO Cellular Component were mainly related to integral components of membrane (GO:0016021) in ILB432, to membrane (GO:0016020) in ILB388 and to extracellular region (GO:0005576) in RRC-1481. The three *C. paspali* isolates did not show significant differences in the percentage of genes in the total secretome in any of the categories of these GO domains.

### Identification of possible pathogenicity factors like proteins in *Claviceps paspali* isolates

Fungal effectors often share certain sequence characteristics such as size, cysteine content, or small motifs identified in fungi. The small secreted proteins (SSP) comprise 65.7% of the ILB388 secretome, 64.5% in ILB432 and 67.3% in RRC-1481 (Table 2). Furthermore, the small rich cysteine proteins (SRCP) in the entire secretome were of 33.2% for ILB388, 32.0% for ILB432 and 31.0% for RRC-1481. Intriguingly, 65% (81 proteins), 65.6% (67 proteins) and 73.4.9% (83 proteins) of the total SRCP in ILB388, RRC-1481 and ILB432 respectively, had no homology with proteins in the Nr NCBI database. We also used effector signature motifs such as the [YW] XC found in several fungal secretome as a criterion for mining effectors from the secretome of *C. paspali* isolates. We identified a total of 53, 63 and 55 proteins with reported fungal effector motifs, where the most abundant motif was Y/F/WxC in all the three isolates (Additional file S3). It was found in approximately 10% of the three *C. paspali* isolates PSP and in approximately 3% of the whole *C. paspali* isolates proteome (Additional file S3). This indicates a 3-fold enrichment of these motifs in PSP and was significant in relation to the complete protein dataset.

**Fig. 1:**
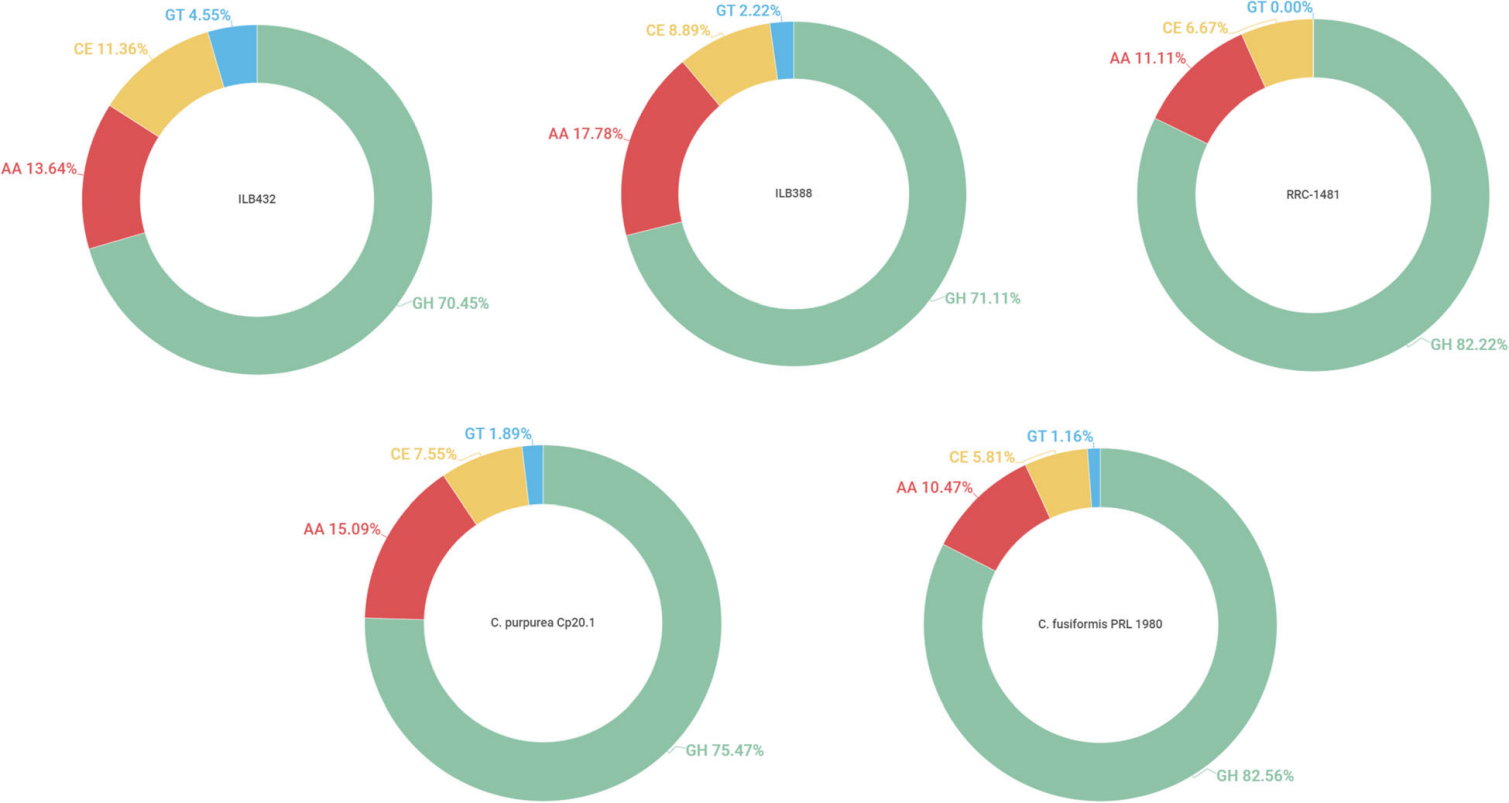
Representation of CAZymes in *Claviceps spp* secretome. A percentage of CAZyme families in each isolate of *Claviceps*. GH = glycoside hydrolase; AA = Auxiliary Activities; GT = glycosyltransferase; CE = carbohydrate esterase

In summary, the dbCAN2 CAZyme annotation of the secretome identified 134 CAZyme-like proteins (45 in RRC-1481, 45 in ILB388 and 44 in ILB432) **(**Fig. 1A), representing 38 CAZyme families (Additional file S4). Only four families were isolate specific, CE5 in ILB432 and CE 3, GH 3, GH47 in isolate ILB388. Among all PSPs identified, at least 98 (33 in ILB388, 32 in ILB432 and 33 in RRC-1481) are suggested to be involved in the degradation of polysaccharides (Additional file S4). Also, the three families with the greater number of proteins in each isolate are predicted to be PCWDEs, these were GH16, GH28 and GH43.

In total, 72, 66, 81 PSP in ILB432, ILB388 and RRC-1481 respectively were BLASTp searched against the Pathogen–Host Interaction (PHI) database, where there are proteins from other plant pathogenic fungi that are involved in pathogenesis and modulate host responses. The predicted pathogenicity-associated genes recovered were classified into four of the nine categories based on the definition of phenotypes in the PHI database [56]. The results of the predictions are summarized in Table 3 and details are presented in Additional File S2. The categories indicating virulence factors, which are the most relevant, are “loss of pathogenicity”, “reduced virulence” and “effectors”. Most of the sequences are classified as reduced virulence in the three isolates (Additional File S2), and there is no significative difference between categories among *C. paspali* isolates.

**Table 3:** Summary of prediction of secretome and pathogenicity related factors in *Claviceps paspali* isolates

Category	ILB388	ILB432	RRC-1481
Total proteins	8012	8243	8122
PSP (% of total proteome)	371 (4.6%)	409 (4.9%)	364 (4.5%)
PSP with BLAST results (% total PSP)	248 (66.8%)	269 (65.8%)	234 (64.3%)
PSP functional annotated (% total PSP)	121 (32.6%)	115 (28.1%)	117 (32.1%)
SSP	244	264	245
Total SCRP	123	127	113
Conserved effectors motifs	53	63	55
Total PHI-base	56	65	81
Total CAZymes	45	54	45
Total effector like proteins (% of total PSP) ^a^	263 (71.1%)	292 (71.4%)	264 (72.5%)
Total predicted pathogenicity factors (% of total PSP) ^b^	325 (87.6%)	353 (86.3%)	316 (86.8%)
Localization of predicted effectors			
Apoplast ^c^	230	239	223
Chloroplast ^d^	13	14	21
Mitochondria ^d^	12	18	8
Nucleus ^d^	32	26	31

^a^ = Proteins with at least one condition defined for effector; ^b^ = Proteins with at least one condition defined for pathogenicity factor prediction; ^c^ = Predicted with ApoplastP; ^d =^ Predicted with Localizer. PSP = predicted candidate secreted protein. SSP = small secreted protein. SCRP = small cysteine-rich protein

The total proteins that were predicted as effector-like by any of the five approaches (PHI-base “effector” category was take into account) ranges between the 71.1% (ILB388), 85.4% of the total secretome in ILB432 to 86.8% in RRC-1481, indicating that almost all the PSP of this species could be predicted as an effector. Of the predicted effector-like proteins, between 31.5 to 32.7% were encoded proteins with no BLAST results. A total of 7 GO terms were over-represented based on Fisher exact test in ILB432 effector-like proteins, and no term was over-represented in ILB388 and RRC-1481 (Additional File S2).

### *Claviceps paspali* repeat sequence content and association with predicted secretome

The three *C. paspali* genomes comprised between 18,6 and 19% of interspersed repeated DNA sequences (Additional file S5). An analysis of the Transposable element (TE) revealed an overrepresentation of long terminal repeat (LTR) elements representing about 62% of the repetitive sequences in the three genomes.

TE identification was used to calculate the average distance (kbp) of each gene to the closest TE fragment on the 5′ and 3′ flanking side (Additional file S5**)**. This analysis was performed for both all PSP and non-PSP gene models. The mean gene TE distance was between 17,53 kbp in ILB432, 23,34 kbp in ILB388, while in PSP were between 15,15 kbp, 19,65 kbps respectively. Based on a Mann Whitney test using all non-PSP genes as control, we determined that PSP were significantly closer to TEs than non-PSP genes in the two analyzed *C. paspali* genomes. However, if we use the distance to LTR as sole input for evaluation, only in ILB432 PSP are significantly closer (*P* < 0.05) to LTR than non-PSP.

### Conservation of predicted secretome across *Claviceps paspali* isolates

**Fig. 2:**
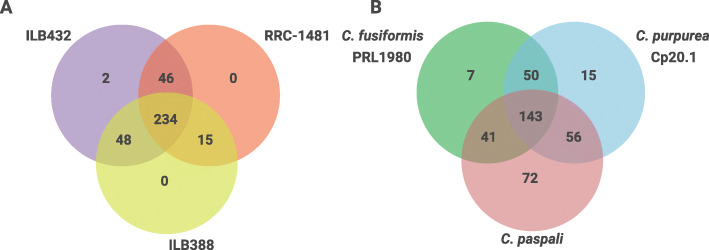
Conservation of PSP based on OrthoFinder results. A Venn diagram showing the number of unique and shared orthogroups in *C. paspali* isolates (A) and across the three *Claviceps* species (B). Species-specific orthogroups are determinate by species-specific and unassigned orthogroups

**Fig. 3:**
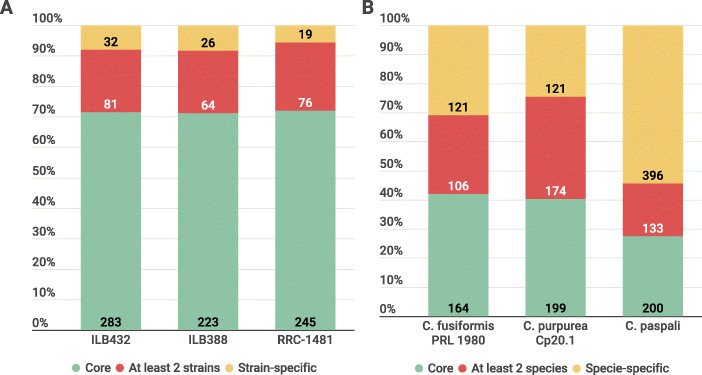
Conservation of PSP based on OrthoFinder results. This figure shows the fraction based in the percentage of unique and conserved proteins within the complete PSP of *C. paspali* isolates (A) and across the three *Claviceps* species analyzed (B)

We determined the unique and conserved proteins across the secretome of *C. paspali* isolates. OrthoFinder was used to identify orthologs between the proteins of three predicted secretome. A set of 345 orthogroups was established for the *C. paspali* isolates, representing a total of 1139 proteins. A total of 234 orthogroups were set as the core secretome and only two orthogroups as isolate-specific (Additional file S6). There was a total of 26, 28 and 19 proteins of ILB388, ILB432 and RRC-1481 respectively that where not assigned to any orthogroups, that could be taken also as secretome isolate-specific proteins. ILB432 and RRC-1481 shared between them 46 orthogroups, ILB388 shared 48 with ILB432 and 15 with RRC-1481 (Fig. 2A). These results indicate that the core secretome in *C. paspali* represent more than 70% of the PSP orthogroups (Fig. 3A).

### Conservation of predicted secretome across *Claviceps* species

We described the probable secretome of *C. purpurea* and *C. fusiformis* (Additional file S7) for comparison with the *C. paspali* PSP. For this comparison, a non-redundant secretome of *C. paspali* was obtained using the CD-HIT algorithm (95% identity) to produce a final list of 729 unique proteins that represent the combined predicted secretome of the three isolates. This non-redundant predicted secretome was compared with the PSP of *C. purpurea* and *C. fusiformis* using OrthoFinder (Additional File S8), resulting in a total of 1614 proteins analyzed (Fig. 2B). For the *Claviceps* species secretome comparison, a total of 143 orthogroups present in all species (core secretome) and a total of 94 species-specific orthogroups (7 in *C. fusiformis*, 15 in *C. purpurea* and 72 in *C. paspali*) were found. Unassigned proteins were 77 (18.7% of the secretome), 71 (14.2%) and 69 (11%) proteins in *C. fusiformis*, *C. purpurea* and *C. paspali* respectively. Interestingly, 54.3% of the secretome of *C. paspali* is unique (Fig. 3B). By contrast, the secretome of the two other *Claviceps* species have a smaller set of unique proteins and a higher set of conserved proteins. Only 30.9 and 24.4%, of the secretome of *C. fusiformis* and *C. purpurea* respectively is unique (Fig. 3B)**.**

### Transcriptomic analysis

To investigate how the fungal genetic program is deployed during a host infection, we applied Illumina RNA sequencing to the *C. paspali - P. dilatatum* pathosystems. Transcriptomic data of two early time points of the fungal infection, 1 and 4 dpi, representing penetration and established infection respectively, were used to generate an overview of the *in planta* expressed portion of the secretome. Based on the fact that ILB432 has the least fractionated genome and belongs to the most common lineage of *C. paspali* affecting *P. dilatatum*, we used its draft genome to analyze the *C. paspali* PSP expressed *in planta* (Additional file S9). To analyze correlation between biological replicates across all conditions a clustering analysis was made (Additional file S10). Hierarchical cluster analysis shows that biological replicates of the same condition are cluster tightly, indicating the reproducibility and correlation between samples.

All genes with a TPM > = 1 in all the replicates in each condition, were considered as expressed in a time point (Table 4). Also, expressed genes were grouped into three categories according to their main expression levels in each condition: low (1 to 10 TPM), medium (11 to 1000 TPM), high (more than 1000). Almost all the protein-coding gene models in *C. paspali* ILB432 were transcribed *in planta* in at least one time point (6861 genes, or 83.2%). In total, 326 (78.7%) sequences of the PSP were expressed during at least one of the two time points, which is smaller than the percentage found for the whole proteome. At 1 dpi is the time point where more PSP (321) and unique PSP are expressed (97). Also, about 30% more predicted pathogenicity factors are expressed during 1 dpi in comparison with 4dpi (Table 4). Approximately 72.9 and 76.2% of effector-like and pathogenicity factor-like proteins are expressed during 1 dpi, while 50.7 and 54.4% at 4 dpi. No significative differences were detected in PSP expression between conditions. Between the first 10 highly expressed genes in both time points evaluated (Additional file S9), 4 genes were PSP and predicted as effector-like, and three of these are shared between 1 and 4 dpi. The first two most expressed genes are the predicted effector-like *CpILB432_gene_07899 and CpILB432_gene_07732*. The second most expressed at 4 dpi is also *CpILB432_gene_07899*. None of these four predicted effector-like proteins has functional annotation or known PFAM domain. To allow for a broad and unbiased evaluation of the fungal transcriptome of these two time points during infection (penetration and colonization), an analysis of differential expression between 1 and 4 dpi conditions was made. Based on this analysis a total of 24 genes with higher expression were found between conditions. Of these, 21 were more expressed during 1 dpi, and three during 4 dpi (Table 5). Three genes that were identified as PSP (*CpILB432_gene* 06873, *CpILB432_gene* 04049 and *CpILB432_gene* 03624), were more expressed during 1 dpi and belong to the 5 genes with more FC with statistical significance (FDR < 0.01) in this comparation. Gene *CpILB432_gene*_06873 was predicted as effector and SCRP, *CpILB432_gene* 04049 was also predicted as effector with [KRHQSA][DENQ] EL motif and *CpILB432_gene* 03624 was predicted as effector, SCRP and with motif [YW]XC.

**Table 4:** Summary of expressed genes during 1 and 4 dpi based on the analysis of expression of RNA-seq mapped to the *Claviceps. paspali* ILB432 genome

Condition	Total expressed genes	PSP	Predicted effector-like proteins	Predicted pathogenicity factors
1 dpi	6772	314	213	269
4 dpi	5492	227	148	192

**Table 5:** Genes differentially expressed in ILB432 during 1 vs 4 dpi

	Gene ID	BLAST Accession	logFC	FDR
**1 dpi UP REGULATED**	*CpILB432_gene_02158*	CCE31305.1|related to major facilitator MirA [Claviceps purpurea 20.1]	−9,923,735,523	7,14E-06
	*CpILB432_gene_05184*	XP_018140586.1|Rad4 transglutaminase-like domain-containing protein [Pochonia chlamydosporia 170]	− 975,031,661	0,00020199
	CpILB432_gene_06873*	CCE27092.1|uncharacterized protein CPUR_00564 [Claviceps purpurea 20.1]	−6,095,858,717	0,0004902
	*CpILB432_gene_03624*	CCE27096.1|uncharacterized protein CPUR_00568 [Claviceps purpurea 20.1]	−5,991,756,785	0,0004619
	CpILB432_gene_04049*	CCE31699.1|uncharacterized protein CPUR_05553 [Claviceps purpurea 20.1]	− 489,987,136	5,74E-05
	*CpILB432_gene_06757*	EWZ78380.1|hypothetical protein FOWG_17350 [Fusarium oxysporum f. sp. lycopersici MN25]	−3,892,808,943	1,30E-08
	*CpILB432_gene_02058*	CCE33483.1|probable SIT1-Transporter of the bacterial siderophore ferrioxamine B [Claviceps purpurea 20.1]	−3,679,279,939	5,62E-10
	*CpILB432_gene_04875*	XP_007813912.1|extracellular serine-rich protein [Metarhizium acridum CQMa 102]	−2,984,494,587	0,00054842
	*CpILB432_gene_05927*	CCE30713.1|uncharacterized protein CPUR_04562 [Claviceps purpurea 20.1]	−2,937,451,378	0,0004619
	*CpILB432_gene_03545*	KDB16458.1|short-chain dehydrogenase/reductase 2 [Ustilaginoidea virens]	−2,875,735,595	1,30E-08
	*CpILB432_gene_03512*	No Blast Hit	−2,849,291,501	5,36E-05
	*CpILB432_gene_05140*	XP_018140533.1|allergen [Pochonia chlamydosporia 170]	−2,844,088,745	5,55E-05
	*CpILB432_gene_02054*	XP_007819099.2|hypothetical protein MAA_02910 [Metarhizium robertsii ARSEF 23]	−2,835,956,686	3,27E-05
	*CpILB432_gene_06755*	CCE32677.1|uncharacterized protein CPUR_06541 [Claviceps purpurea 20.1]	−2,747,141,458	6,99E-06
	*CpILB432_gene_00516*	CCE29318.1|related to O-methylsterigmatocystin oxidoreductase [Claviceps purpurea 20.1]	−2,699,405,679	5,36E-05
	*CpILB432_gene_03325*	KZZ93778.1|hypothetical protein AAL_05494 [Moelleriella libera RCEF 2490]	−2,679,797,919	7,74E-05
	*CpILB432_gene_07758*	KHN97956.1|hypothetical protein MAM_04345 [Metarhizium album ARSEF 1941]	−2,359,145,346	5,36E-05
	*CpILB432_gene_02316*	KZZ94274.1|NAD(P)-binding domain protein [Moelleriella libera RCEF 2490]	− 235,850,479	5,55E-05
	*CpILB432_gene_00162*	No Blast Hit	−2,266,379,409	3,27E-05
	*CpILB432_gene_08111*	OAA32324.1|Thioredoxin-like protein [Moelleriella libera RCEF 2490]	−2,210,366,996	0,00026004
	*CpILB432_gene_02137*	RZR60711.1|hypothetical protein I1G_00000288 [Pochonia chlamydosporia 123]	−1,993,353,249	0,00041294
**4 dpi UP REGULATED**	*CpILB432_gene_03143*	CCE31647.1|uncharacterized protein CPUR_05500 [Claviceps purpurea 20.1]	212,350,458	5,36E-05
	*CpILB432_gene_03342*	No Blast Hit	2,235,801,505	0,00038791
	*CpILB432_gene_02170*	KFG81740.1|Amino-acid permease inda1 [Metarhizium anisopliae]	3,083,272,028	5,06E-06

* = predicted as PSP

Validation of gene expression by RT-qPCR was not done based on previous studies that shown extremely close correlation between RT-qPCR and RNA-seq results [57–61]. Also, our biological samples are highly similar within treatments and clearly distinct between treatments (Additional File S10).

## Discussion

### Claviceps paspali genome

This paper reports, for the first time, an analysis of whole genome sequences for multiple isolates of *C. paspali* assembled with short-reads. A high proportion (> 95%) of single copy BUSCO genes were recovered showing a high completeness in all three analyzed genome assemblies. All *C. paspali* isolates had highly compact genomes, with sizes of 28–29 Mb. This is similar to other *Claviceps* spp. genomes, that range between 28.9 and 52.3 Mb [54], but smaller than the average genome size of plant pathogenic ascomycetes (39.4 Mb) [62]. Approximately 23%, of the genome was repeated content, where TE was the most prominent family. This is similar to other fungi where TEs comprise1 to 25% of the genome [63].

We also annotated for the first-time a *C. paspali* genome using RNA-seq data. The genomes of the three *C. paspali* isolates were very similar regarding protein coding genes. The ILB432 and RRC-1481 genome assemblies were smaller than the ILB388 assembly. Even so, these differences in genome and TEs content are not significative and could represent a bias in sequencing technology. It is known that short reads could lead to sub-representation of genome size problems by bad resolution of complex regions like repetitive gene sequences or transposable elements [64]. These issues could be solved with long-reads sequencing (like PacBio or Oxford Nanopore) and should be addressed in future in these species.

### The *Claviceps paspali* predicted secretome

The repertoire of PSP of these *C. paspali* isolates helps understand the pathogenic process, due to their putative roles in penetration, host tissue degradation and host immunity subversion [18, 65–67]. Several studies have reported that the size of fungal secretome correlates with lifestyle [18, 68–70]. Furthermore, the types of secreted proteins during host infection depend on the pathogenic lifestyle of the fungus. Necrotrophic fungi secrete plant cell wall-degrading enzymes or toxins to kill their host cell, whereas biotrophic fungi like *C. paspali* secrete proteins to avoid or suppress host defense responses to keep their host cells alive thus maintaining a long-term feeding relationship [71]. The reported host range of C. paspali is at least 19 species of the Paspalum genus [4], but its secretome is about 4.5–4.9% of total proteome. This predicted secretome was in accordance with data from several other facultative biotrophs where approximately 5 to 10% of the proteome were PSP [18]. This suggests that the broad host species range was not achieved by acquiring many secreted proteins in C. paspali. Furthermore, small secretome may minimize the potential for triggering plant immunity following detection of secreted proteins [44].. Also, PSP in *C. paspali* could encoded about 400 novel proteins that lack of known functional domains and homology to known proteins, similar to other fungi with the same nutritional lifestyle [18, 70, 72]. These proteins could be involved in novel strategies specialized in both for infection and evasion of immunity in species of the genus *Paspalum*.

Similarities between these isolates in their relative abundances of functionally annotated genes could suggest a conserved infection strategy. Hydrolase activity (GO:0016787) was the most common molecular function of the predicted secretome for each *C. paspali* isolates, similar to secretomes in several pathogenic fungi [42, 69, 73], including *C. purpurea*-rye interactions [49].

It has been reported that PSP could be located in more repeat-rich regions and TE could be associated with the loss or gain of a particular gene responsible for rapid evolution, including gain and loss of pathogenicity-related genes in these isolates [i.e: 84, 85, 97, 98]. In *Claviceps* genus there are reports of this in *Claviceps* and *Pusillae* sections [74]. Our data shows that TE are significantly close to PSP in the two analyzed C. *paspali* isolates. This difference between our report and previous reports for RRC-1481 [74] could be due the difference in structural annotations used for analysis and prediction method for PSP. Also, PSP in ILB432 (and not in ILB388) were found to be significantly closer to LTRs compared with other genes. These differences could support the theory of differentiation of lineages in the *C. paspali* species. The proximity of LTR to secreted proteins might cause duplication or loss events that could lead to speciation in this lineage. Further research should be made into this matter to address this point.

### Approach to the effector-like proteins repertoire in *Claviceps paspali* species

We identified a variable number of predicted effector-like coding genes among *C. paspali* isolates using different criteria. During the penetration phase, *C. paspali* grows mainly intercellularly [2]. Unlike *C. purpurea* [75], *C. paspali* has not been documented to produce specialized intracellular structures, suggesting that interaction with the plant may be predominantly apoplastic. This was in consonance with our results: between 54 and 63% of the PSP were predicted as secreted to the apoplast with ApoplastP. Thus, *C. paspali* could be a fungal pathogen like *Cladosporium fulvum*, *Zymoseptoria tritici*, *Leptosphaeria maculans* and *Venturia inaequalis*, which colonize plants extracellularly and rely on effectors to target basal apoplastic host defense components [76, 77].

Reactive oxygen species, calcium oscillations and the synthesis of plant defense molecules, such as phytohormones, are produced in the chloroplast and are essential providers of redox resources to fight pathogen attacks. These functions are key in the early defense of the plant [30, 78]. In our case, almost 11% of the total PSP in the three isolates were predicted to have an action in the chloroplast. Interestingly, no effector-like proteins were found to be involved in phytohormone synthesis and this is an aspect of study that should be further explored.

Within the fungal secretome, SSPs and SCRP have been widely studied for their role in pathogenesis, particularly functioning in the apoplast [18, 77, 79]. The three isolates of *C. paspali* had approximately 65 and 33% of SSP and SCRP respectively, more than the average SSP (52%) and SCRP (23%) contents in plant pathogen fungi [22] but similar to values obtained in biotrophs [70]. This gives weight to the theory that biotrophs encode more and more diverse effector-like SSPs to suppress host defense compared to necrotrophs, which generally use cell wall degrading enzymes and phytotoxins to kill hosts [18, 72, 80]. Approximately more than half of the SCRP in each isolate lack homology to proteins in other species, like was reported before for fungi and oomycete plant pathogens [18].

All searched for conserved effector motifs were present in *C. paspali* isolates PSP. Based on this search we identified that the motif [YFW] xC is the most abundant in the *C. paspali* PSP. This motif was first discovered in the barley powdery mildew obligate biotroph fungus *Blumeria graminis* [81] and later in rust fungi [82]. Typically, this motif is in close proximity to the signal peptide, but like with rust fungi, [82] this motif was found dispersed and no restricted to the N-terminus in *C. paspali* isolates. The occurrence of this motif in PSP is more than three times higher compared to the whole proteome in *C. paspali* isolates. Based on this we suggest that this could be a useful motif for effector candidate prediction in *C. paspali*.

PSP, matched with known effectors in the PHI-base, were included in the set of effector-like candidates. In the three isolates there is a homologue of the *LysM1* a *Penicillium expansum* effector protein. Effectors carrying LysM domain have been identified in numerous pathogenic fungi and their role in the first stages of infection has been established, based on chitin sequestration [83]. In *C. purpurea*, deletion of gene *Cp8623,* a LysM carring secreted protein, showed a decrease in virulence. Therefore, this type of protein could be important but not determinant in the development of Ergot disease.

### Plant cell wall degrading enzymes in the predicted secretome of *Claviceps paspali*

Most biotrophic fungi degrade plant tissues by producing a set of enzymes specifically focused on plant polysaccharide degradation [18, 84]. These are critical for pathogenicity because many of them break down the physical barrier to the host tissue, and their breakdown products include sugar monomers that could be food sources. Pectin (a polymer of mainly D-galacturonic acids) hydrolysis has been proved to be an important step for fungal penetration in monocots [85, 86]. The fact that pollen tubes show pectinolytic activity [87], could be taken as evidence to the hypothesis that *Claviceps* species mimic pollen tube and adapt their lifestyle to the pollen tube/plant tissue system [5].

The relevance of pectin degradation for *C. paspali* colonization could be consistent with our findings where two of the CAZyme families with the highest number of genes were the GH28 (4 genes) and GH43 (4–5 genes) families. Both families could be associated to pectin degrading enzymes. A common match in the three isolates in the BLASTp against the PHI-base was the *CPPG2* gene of *C. purpurea* that encodes a endopolygalacturonase involved in the degradation of the pectin present in the style and ovary tissue of rye [45], and a pectin methylesterase *BCPME1* of *Botrytis cinereal* who also is a key virulence factor in this fungus [88]. This could lead to the conclusion that the *C. paspali* infection process is similar to the infection process of *C. purpurea* on rye flowers where in the early stages of infection pectin degradation and polygalacturonase activity represent a pathogenicity factor.

Also, the secretome of *C. paspali* contains enzymes involved in chitin degradation, which is an important component of the fungal cell wall, and one of the most studied molecules that activate plant defenses [25, 89]. Based on BLASTp results to the PHI-base, in the three isolates there were PSP homologous proteins matching a known effector GH18 chitinase like *CHT42* of *Trichoderma virens.*

### An approach to host speciation related to the predicted secretome in the *Claviceps* species

The three analyzed species have completely different host ranges. While *C. purpurea* causes ergot of wheat, rye, barley, oats, and many other host species [90], *C. fusiformis* is restricted to pearl millet (*Pennisetum glaucum*) and buffel grass (*Pennisetum ciliare*) [15, 91], and *C. paspali* is a pathogen of grasses in the genus *Paspalum* [2]. It has been proposed that gene loss/gain were the hallmark of jump events to new hosts [92]. The unique and conserved proteins identified across the secretome of *Claviceps* species could lead to a better understanding of the important players for virulence and host specificity. Genome comparisons of *C. paspali* with the related Ergot causing fungi *C. purpurea* and *C. fusiformis* revealed that 27–43% are conserved PSP in three species sequenced so far and we call them core secretome.

Among the few functionally characterized pathogenicity factors in Ergot pathogens, the *CPPG2* gene [45] was shared among all *Claviceps* species and might constitute a core virulence factor for the establishment of the disease or for enhancing pathogen fitness. All Ergot species analyzed had proteins in the orthogroup OG0000053, formed by orthologs of the *CPPG2* gene. However, besides this enzyme, no other *Claviceps* spp. proved pathogenicity factor were part of the core secretome. Other characterized *C. purpurea* predicted secreted pathogenicity factor like *CPPG1* [45] showed no shared orthologs in any of the *C. fusiformis* and *C. paspali* secretome. Species-specific PSP may explain the observed differences in host adaptation among species and isolates [42, 93]. However, the secretome of *C. purpurea* has the smallest fraction of unique proteins which is not in consonance to its broad host range of this species. This could suggest that relaxed selection pressure may have led to the reduced effector coding gene content in *C. purpurea* species. The effector repertoire is probably highly redundant [49], and some effectors may no longer be essential since there is no genetic resistance in the host. This suggests that the broad host range is not achieved through the acquisition of a large number of unique secreted proteins.

The distribution of CAZymes categories was similar among the three Ergot pathogens analyzed. GH28, GH16 and GH28 were between the most abundant families in the three species (Additional file S11). This could suggest the importance of these families and the degradation of pectin and hemicellulose in the infection process of this species, as was previously reported [5].

### Predicted secreted proteins expressed *in planta* during *Paspalum dilatatum* infection

**Fig. 4:**
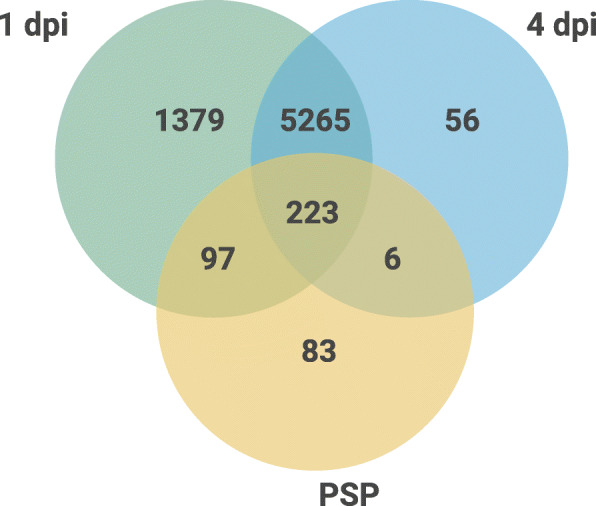
Venn diagram of expressed genes of C. paspali ILB432 during each time point of infection. Identification of unique and conserved expressed PSP of *C. paspali* ILB432

This is the first time that RNA-seq data is reported for a *C. paspali*-host interaction. Although many genes may not be detected in the early stages of infection because the amount of biomass in the fungus increases over time, as does the number of sequential readings originating from the fungus, we found that most of the predicted genetic models are expressed at 1 dpi. This is probably due to the imperative need to establish early growth conditions of hyphae in the stigma and that very few sequences were detected exclusively at 4 dpi (Fig. 4). At least 326 PSP of ILB432 were expressed during first stage of infection of *P. dilatatum* cv Estanzuela Chirú. This represents about 79.7% of the total secretome. Nevertheless, no significative differences were established between expression of PSP and the full set of proteins. This could suggest that *C. paspali* requires a maximum capacity for host manipulation during intracellular colonization and that biotrophic hyphae provide a major interface for effector delivery to host cells. Expression of the predicted secretome and effectors shows the same tendencies as the whole genome where there was a higher expression at 1 dpi. As we can see after the infection has taken place (4dpi) the fungus regulates the expression of several of PSP. This unique profile expressed proteins during 1 dpi could be important for the first stage of penetration and colonization but not for maintaining and stabilizing the infection. Even so, these results have to be taken with caution because of the absence of a basal fungal condition control like *C. paspali* axenic culture data. We cannot make claims about these genes being pathogenicity factors important for infection or if they are highly expressed housekeeping genes essential for basic biological functions. However, differences found suggest an interesting lead for pathogenicity factors recognition.

Our results, based on predictions from DNA sequence and RNA-seq analysis, show that three probable PSP and effectors have mayor FC during 1 dpi compared to 4 dpi. These three genes: *CpILB432_gene* 06873, *CpILB432_gene* 04049 and *CpILB432_gene* 03624 had homology to three uncharacterized *C. purpurea* proteins. These results are similar to those obtained by Oeser [49] who found that during *C. purpurea*-rye interaction, the fungus expressed several effector candidates that were included in the 150 most highly expressed genes during infection, several of which did not have homology to any known proteins. Five of these even belonged to the top ten highly expressed genes. These results are also similar to our data, where 4 of the first 10 highly expressed genes in both time points evaluated were predicted as effector-like, and three of these are shared between 1 and 4 dpi. However, the role of these PSP and pathogenicity factor-like proteins in *C. paspali-Paspalum* pathogenesis, remains largely unanswered and further work should address this topic like knock-out stains and comparations with axenic culture RNA-seq data.

## Conclusions

In this study we present an annotation for three draft genome sequences of *C. paspali* isolates that belong to two different lineages inside *C. paspali* species using a combination of RNA-seq, manual gene curation and comparative genomic techniques. The combination of genome and transcriptome sequencing allowed for data-driven gene prediction and comparative genomics with other public available genomes of Ergot disease fungal species. Our results, based on predictions from DNA sequence, showed that *C. paspali* isolates from both lineages share the main signatures and protein families in the predicted secreted proteins. The abundance of pectinolytic enzymes and the presence of chitin-degrading enzymes in the genome and secretome of *C. paspali* implies that they could be part of its pathogenic system, causing Ergot disease. The small predicted secretome size of *C. paspali* and the absence of similarity to experimentally validated effectors from other pathogens strongly suggest that *C. paspali* employs largely novel mechanisms to induce susceptibility in its host plants. This genome data and predicted secretome repertoire provides insights to design hypotheses about candidate host range determinants in the *Claviceps* genus and suggests details to direct biological experimentation. The analyzed genomes of *C. paspali* along with other *Claviceps* species phylogenetically closely related to each other enabled us to zoom in and further characterize secreted proteins with potential roles in the infection process.

## Methods

### Sequence information

For *C. paspali* isolates analysis (Table 6), draft genome sequence of isolate ILB432 was downloaded from NCBI Acc. GCA_013168865.1 [54] and RRC-1481 form NCBI Acc GCA_000223175.2 [94].

**Table 6:** Information about the isolates used in this study

Species	Isolate	Host	Protein Source	Genome reference
*C. paspali*	ILB388	*P. plicatulum*	This work	This work
*C. paspali*	ILB432	*P. urvillei*	This work	[53]
*C. paspali*	RRC-1481	*Paspalum spp.*	This work	[94]
*C. fusiformis*	PRL 1980	*Secale cereale*	http://csbio-l.csr.uky.edu/endophyte/	[94]
*C. purpurea*	20.1	*Pennisetum typhoideum*	NCBI Acc PRJEA76493	[94]

For identification of core predicted secretome between *C. paspali* and two other related species in the genus, proteins were downloaded from NCBI Acc PRJEA76493 for *C. purpurea* isolate 20.1 and *C. fusiformis* PRL1980 was downloaded from http://csbio-l.csr.uky.edu/endophyte/

### Isolate sampling and genome assembly

*C. paspali* ILB388 isolated from *P. plicatulum* [52] was obtained from the “Laboratorio de Bioproducción” at INIA Las Brujas Fungal Collection (ILB). Vegetative mycelium was harvested form cultures kept in *Claviceps* medium [95] for 1 wk. at 26 °C. Fungal genomic DNA was extracted using Quick-DNA™ Fungal/Bacterial Kit (Zymo Research, San Diego, USA) following the manufacturer’s instructions.

For genome sequencing, DNA libraries of 500 bp inserts were generated with TruSeq Nano DNA Kit (Illumina) and 150 bp paired-end (PE) sequenced with Illumina HiSeq2500 platform (Illumina, San Diego, CA). was performed at Macrogen Inc., Seoul, South Korea service.

For data analysis, low quality and adapter sequences were trimmed via Trimmomatic v0.36 [96]. High quality PE were then assembled with SPAdes 3.5.0 using k-mer lengths based on read length (kmer of 21, 33 and 55 bp for 151 bp reads, with -careful option) [97]) and contigs shorter than 200 bp were removed. All assemblies from *C. paspali* were checked with Quality Assessments Tool (QUAST 4.5, [98]) using –fungus,--split-scaffolds and -min-contig = 200 parameters. Assembly completeness was also analyzed with Benchmarking Universal Single Copy Orthologs (BUSCO v5.0.0 [99];) using –genome mode and --augustus_parameters = ‘--species = *Fusarium graminearum*’ and ascomycota_odb10 database.

### Claviceps paspali in planta RNA-seq data

For RNA fungal expression analysis during infection (*in planta* analysis), fungal spores were collected according to Luttrell (1977) and prepared for plant inoculation according to Oeser et al. (2017). A concentration of 1 × 10^6^ conidia/ml suspension was spread over stigmas of *P. dilatatum* plants cultivar Estanzuela Chiru one day post anthesis and incubated in a sealed, humidified tray at room temperature. Two biological replicates of the experiment were conducted in the greenhouse facility at INIA Las Brujas research station. Stigma infected samples were collected from plants at 1- and 4-days post-inoculation (dpi) for RNA extraction. The criteria for the selection of the two time points was considering the phase of penetration of the fungus (1 dpi) and when the colonization has already taken place on the ovary (4 dpi) [2, 12]. Each biological replicate represents a pool of 400–450 stigmas of one simple plant in order to reach RNA minimum concentration.

Samples were ground to a fine powder in a RNAse-free mortar and pestle, pre-cooled with liquid nitrogen. Total RNA was extracted using RNAeasy Plant isolation kit (Qiagen, Germany). RNA quantity and quality control were assessed using an Agilent 2100 Bioanalyzer (Agilent Technologies, Palo Alto, CA, USA). Polyadenylated mRNA was isolated from the total RNA and cDNA libraries were prepared using a Sure Select Strand-Specific RNA Library Prep mRNA kit (Illumina, San Diego, USA) following the manufacturer’s instructions. Then, constructed libraries were sequenced at the 2 × 150 bp PE read mode with Illumina HiSeq3000, performed at AgriBio, Centre for AgriBioscience, Melbourne, Australia. For raw RNA-seq data, Illumina adaptors and bases having a quality score value less than 25 were trimmed from both ends (final minimum size allowed after trimming of 75 bp) using Trimmomatic v0.36.

### *Claviceps paspali* genome gene predictions

For *C. paspali* ILB388, ILB432 and RRC-1481 FunGAP pipeline [100] was used for structural annotation of genome. This pipeline uses AUGUSTUS [101], MAKER [102], and BRAKER [103] gene model prediction algorithms. Also, both *in planta* condition (1 and 4 dpi) RNA-seq data filtered reads were used for transcript evidence for gene predictions. A *Fusarium graminearum* gene model was selected for AUGUSTUS, with *C. purpurea* isolate 20.1 (GCA_000347355.1), *Metarhizium rileyi* isolate Cep018-CH2 (GCA_007866325.1) and *Pochonia chlamydosporia* isolate 123 (GCA_000411695.2) as sister species.

### Prediction of secreted proteins in *Claviceps* isolates

For the determination of a probable set of secreted proteins (PSP) in the five isolates of Claviceps spp., prediction of signal peptides (SP), was carried out with Phobius web server [104] and SignalP v5 [105]. To exclude membrane proteins, the TMHMM 2.0 web server (max. 1 PredHel within first 60 amino acids) [106] was used in combination with transmembrane domain (TM) prediction by Phobius web server (no TM predicted). Note that TM domain predictions by TMHMM within the last 70 amino acid residues of the N-terminus of a protein sequence were not considered as the tool can sometimes predict signal peptides as false-positive TM domains. To exclude sequences that have a SP but remain in the endoplasmic reticulum, all predicted secreted sequences were scanned for retention motifs from the PROSITE database (PS00014 ER_Targeting) with the ScanProsite web server [107]. Web versions of WolfPSort (organism type: fungi) [108], TargetP 2.0 [109], and ProtComp v9.0 (Softberry, USA) were used to predict the subcellular localization of sequences. The PredGPI prediction server (General model) [110] was used to predict secreted proteins that contain a GPI anchor.

At least two tools were used for inferring the presence of signal peptide, TM domain and subcellular localization, so resulting decisions were made based on the majority rule.

### Functional annotation of sequences

All the predicted protein-coding genes of the three *C. paspali* isolates were functionally annotated using BLASTp [111] against the non-redundant (nr) database of the National Center of Biotechnology Information (NCBI) and classified using InterProScan v.5.19 [112]. Pfam protein families, InterPro domains, gene ontology (GO) terms classification and metabolic pathways (KEGG) were recovered from the BLAST identified proteins using Blast2GO annotation system [113]. BLASTp was made with cut-off E-value of ≤1e-5. Mapping and annotation were performed on Blast2GO using default parameters.

### Analysis of repeats sequences

Determination of repeat sequences could help to explain divergence and speciation in fungal genomes. For this matter*,* the three *C. paspali* repeated sequences present in their genome were predicted by de novo and homology-based methods following the Berriman et al. (2018) protocol. The de novo transposon libraries were constructed with the de novo software RepeatModeler (http:// repeatmasker.org/RepeatModeler/) and LTRharvest [114]. Homology-based libraries were constructed with TransposonPSI (http://transposonpsi.sourceforge.net). Repeat libraries were classified using RepeatClassifier (part of the RepeatModeler software) and merged using USEARCH v7 [115] to cluster the candidate sequences with ≥80%. The genomes where then analyzed using this non-redundant library using RepeatMasker (http://repeatmasker.org).

Results were then used to calculate the average distance (kbp) of each gene to the closest transposable element (TE) fragment on the 5′ and 3′ flanking side, TEs overlapping with genes were also considered as well as a distance of 0 kbp. This was performed for all the gene models and proteins of interest with customized scripts available upon request.

Statistical tests were performed with a non-parametric Mann Whitney using the mean TE closeness of PSP and all the non-PSP as control. RRC-1481 was not considered for this analysis because of the heavily partitioned available genome.

### Pathogenicity factors prediction pipeline

Effector-like proteins in each isolate were identified from the predicted secreted proteins (PSP) that fulfill at least one of the following criteria previously described for classically secreted effector-like proteins in fungi by Sonah et al. [66] with some modifications: (I) small secreted proteins (SSPs), defined as those with sequence length less than or equal to 300 amino acid residues; (II) SSP with cysteine-richness (SCR). Considered cysteine-rich proteins in this study are those which contain at least 4 cysteine residues and have greater than 5% of their total amino acid residues as cysteines; (III) proteins with known fungal or oomycetes effector motifs (DEER, RXLR, RXLX [EDQ], [KRHQSA][DENQ] EL, [YW] XC and RSIVEQD) that were assessed using the FIMO package in MEME program suite [116] with E-value cut-off of ≤1e-4; (IV) EffectorP 2.0 [67] prediction tool was also considered for effector prediction.

Proteins with homology to known virulence/pathogenicity factor from the PHI-base (http://www.phi-base.org/) were also included in the set of candidates. DbCAN2 (http://cys.bios.niu.edu/dbCAN2/) was used to identify all carbohydrate-active enzymes (CAZymes) in the predicted secretome with an e-value cutoff of 10–10 using HMMER [117], Hotpep [118] and DIAMOND [119] and Plant cell wall degrading enzymes (PCWDE) were identified based on Lo Presti et al. [18].

Prediction of localization targeting of PSP after secretion was identified using ApoplastP [77] and LOCALIZER [120].

### Comparative analysis of orthologous gene families

The orthologous groups among the three species, C*. paspali, C. purpurea and C. fusiformis* were identified with the help of OrthoFinder [121]. Orthologous gene pairs were considered based on the amino acid sequence similarity sharing up to 50% of the total length of the shorter gene being analyzed (BLASTp, threshold E-value ≤1e-5). The PSP were classified as species-specific if there was no orthologs of these proteins in the other species being considered. Also, in *C. paspali* isolates orthologues comparisons, the PSP were classified as isolate-specific when there were no orthologs in other *C. paspali* isolate. We defined core as the full set of predicted proteins belonging to orthologous groups present in all three species or isolates.

### RNA-seq expression analysis

Based on the fact that the relative expression of the secreted proteins can be related to the space-time function, the quantification of gene expression was carried out. Filtered reads of both condition (1 and 4 dpi) were aligned to the ILB432 reference genome with STAR [122] using the previously obtained GFF3 annotation file and RSEM [123]. A statistical analysis of the expression data was performed by EdgeR [124]. The DEGs were identified using the following conditions: − 2 > fold change > 2 and FDR (*P* < 0.05).

Clustering analysis for correlation between samples were made using PtR script of the Trinity v2.11.0 package.

## Supplementary Information

**Additional file S1.** Genome annotation report of C. paspali isolates. Report of genome annotation of isolates ILB388 and ILB432 based on the FunGAP pipeline.

**Additional file S2.** Functional annotation of C. paspali isolates PSP. Functional annotation and enriched GO terms of PSP, pathogenicity factors and effector-like proteins in C. paspali isolates.

**Additional file S3.** Effector motif [YW] xC in proteins of C. paspali isolates. Detailed description of results based in FIMO motif search of [YW] xC motif in C. paspali proteins.

**Additional file S4.** CAZyme present in each predicted secretome of five Claviceps isolates. Detailed results of dbCAN2 search of CAZymes based on the predicted secretome of each of the five isolates of Claviceps spp. compared in this study.

**Additional file S5.** Closeness of coding genes in C. paspali isolates to repeated sequences. Closeness of secreted coding genes and other genes annotate to repeated sequences in genomes of C. paspali isolates ILB388 and ILB432

**Additional file S6.** Orthofinder results in C. paspali isolates. Orthofinder results based on the PSP of each C. paspali isolate used in this study

**Additional file S7.** Secretome and pathogenicity factors predicted in Claviceps spp. Summary of secretome and pathogenicity factors predicted in isolates of *C. purpurea* and *C. fusiformis.*

**Additional file S8. **Orthofinder results based on Claviceps species PSP. Orthofinder results based on the PSP of *C. purpurea* and *C. fusiformis* isolates used in this study

**Additional file S9. **Expression results during infection process of C. paspali in *P. dilatatum* stigma-style. Mapping results, gene ID, and expression values in TPM of all the protein coding genes present in C. paspali ILB432

**Additional file S10.** RNA-seq sample correlation analysis. Clustering analysis of biological replicates between conditions.

**Additional file S11.** Representation of CAZymes in each isolate of Claviceps analyzed in this work. Hierarchical clustering and heatmap of CAZymes families in each isolate of Claviceps.

## Data Availability

The genomic sequences and RNA-seq datasets generated during the current study are available at NCBI BioProject repository, under accession number PRJNA625338. Genomic sequences from ILB432 can be accessed from NCBI Acc. GCA_013168865.1, ILB388 from NCBI Acc GCA_000223175.2 and RRC-1481 from NCBI Acc GCA_000223175.2. *C. purpurea* 20.1 and *C. fusiformis* PRL1980 protein datasets were download from NCBI Acc PRJEA76493 and http://csbio-l.csr.uky.edu/endophyte/ respectively. Structural and functional annotations of *C. paspali* isolates obtained in this study are available from the corresponding author upon reasonable request.

## Abbreviations

bp: base pair
BUSCO: Benchmarking universal single-copy orthologs
DEG: differentially expressed genes
Dpi: days post infection
ETI: effector-triggered immunity
FC: fold-change
FDR: False discovery rate
GO: Gene Ontology
kbp: kilo base pair
n/a: data not available
Nr NCBI: NCBI nonredundant protein database
PAMP: pathogen associated molecular patterns
PCWDE: plant cell wall degrading enzymes
PE: Paired-end
PSP: probable secreted proteins
PTI: PAMP-triggered immunity
SCRP: small cysteine-rich proteins
TE: Transposable element
