# Supplementary material for: Genome-wide analysis of Claviceps paspali: insights into the secretome of the main species causing ergot disease in Paspalum spp
Source: BMC Genomics. 2021 Oct 26;22:766. doi: 10.1186/s12864-021-08077-0 (PMC8549174; doi:10.1186/s12864-021-08077-0)

**Supplementary Table S1. FunGAP report for transcriptome reads assembly**

| **Attributes** | **ILB3432** |  | **ILB388** | **RRC-1481** |
| --- | --- | --- | --- | --- |
| Number of assembled contigs | 32,874 |  | 22,012 | 32,712 |
| Number of contigs > 1 kbp | 9,468 |  | 6,397 | 9,514 |
| Total transcript size (Mbp) | 27,986,155 |  | 17,948,675 | 28,094,234 |

**Supplementary Figure S1. ILB432 transcript length distribution**


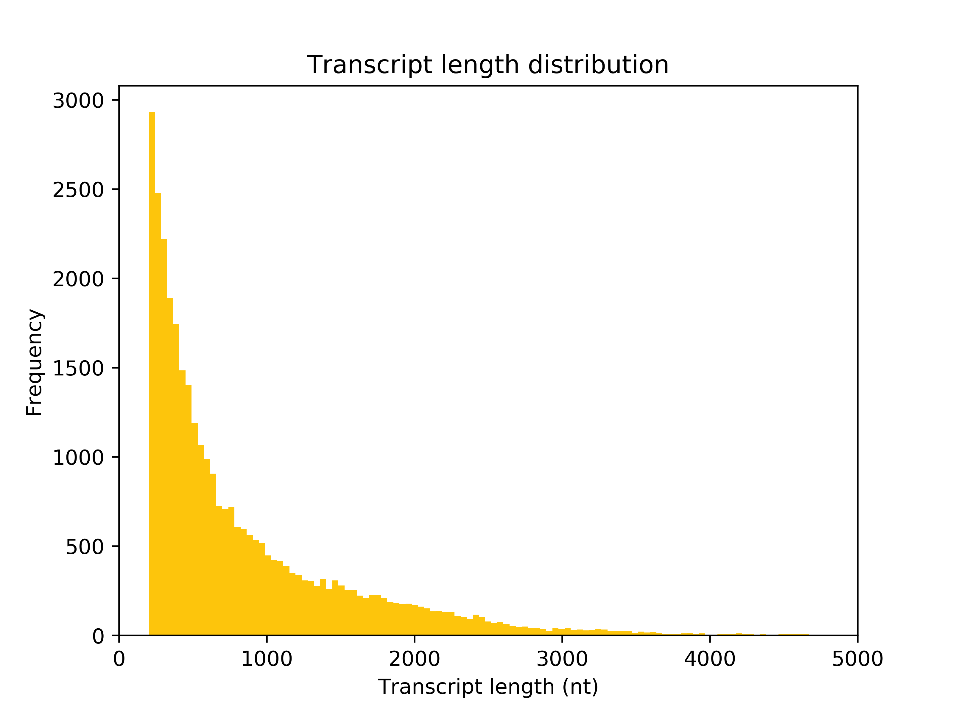


**Supplementary Figure S2. ILB432 protein length distribution**


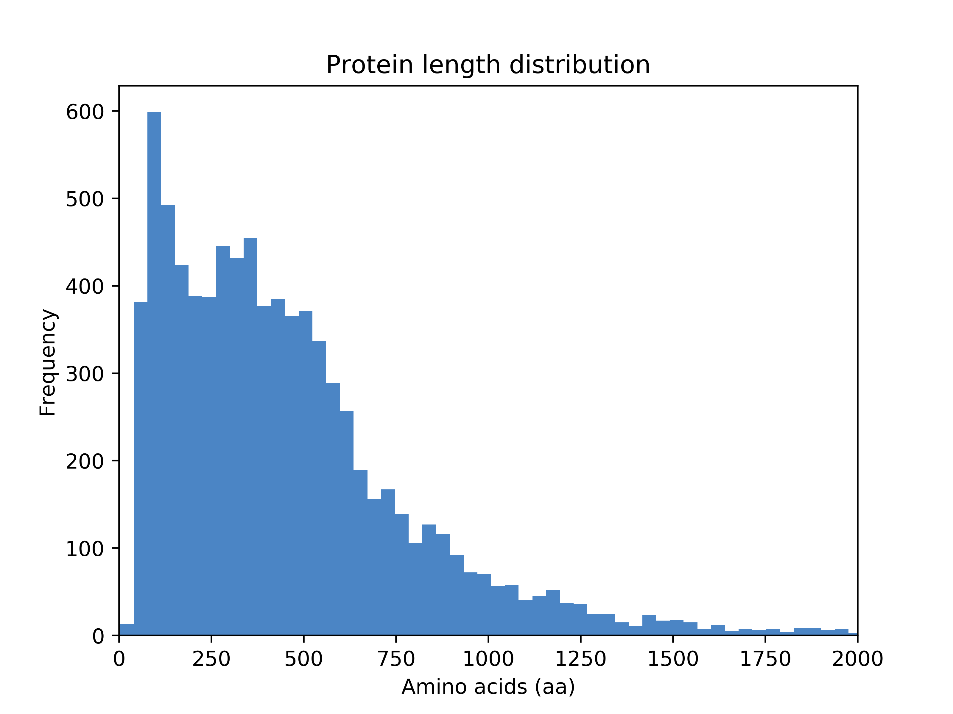


**Supplementary Figure S3. ILB388 Transcript length distribution**


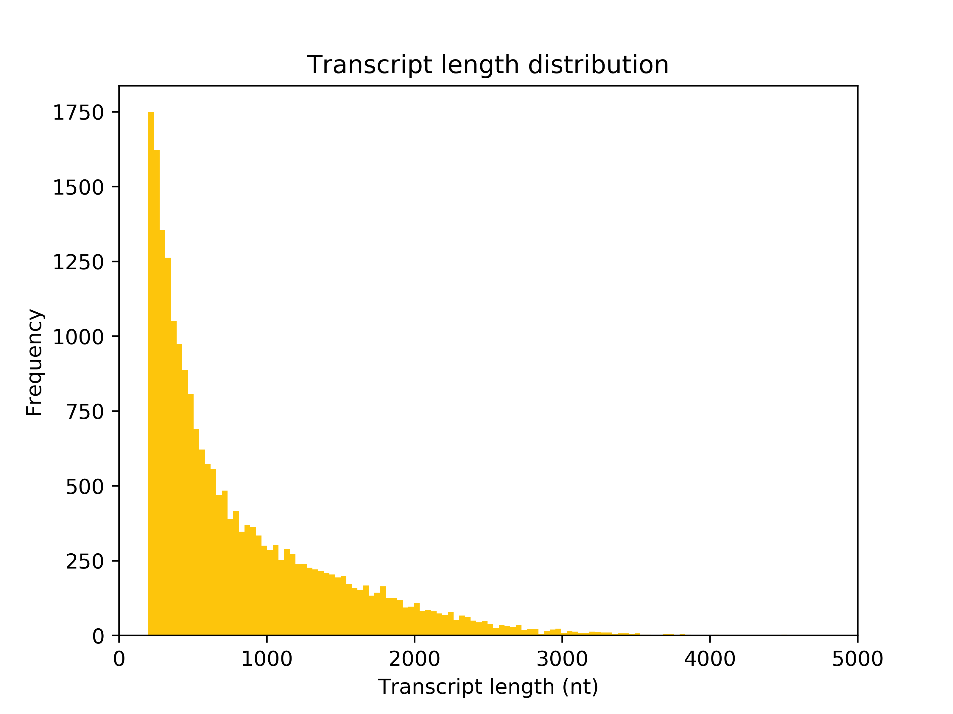


**Supplementary Figure S4. ILB388 protein length distribution**


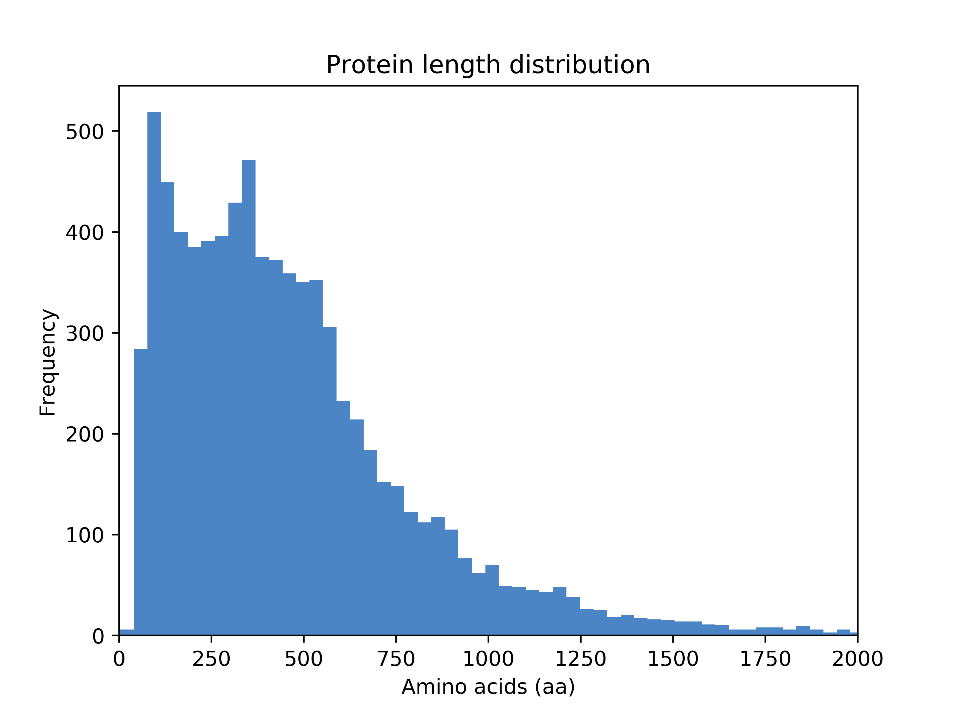


**Supplementary Figure S5. RRC-1481 Transcript length distribution**


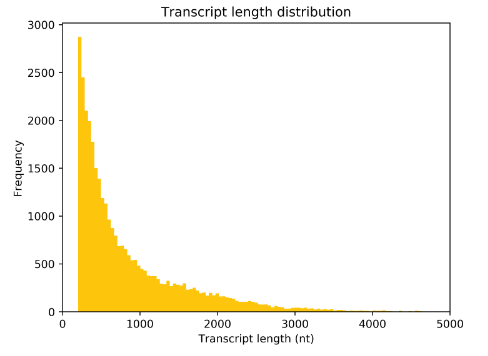


**Supplementary Figure S6. RRC-1481 protein length distribution**


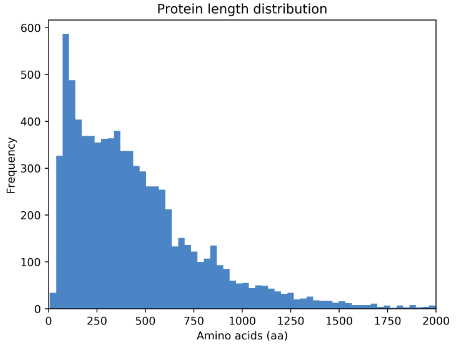

Supplement: Supplementary file 1 — Additional file S1. Genome annotation report of C. paspali isolates. Report of genome annotation of isolates ILB388 and ILB432 based on the FunGAP pipeline. [file 12864_2021_8077_MOESM1_ESM.docx]
