# Supplementary figures and images for: Genome-wide analysis of Claviceps paspali: insights into the secretome of the main species causing ergot disease in Paspalum spp
Source: BMC Genomics. 2021 Oct 26;22:766. doi: 10.1186/s12864-021-08077-0 (PMC8549174; doi:10.1186/s12864-021-08077-0)

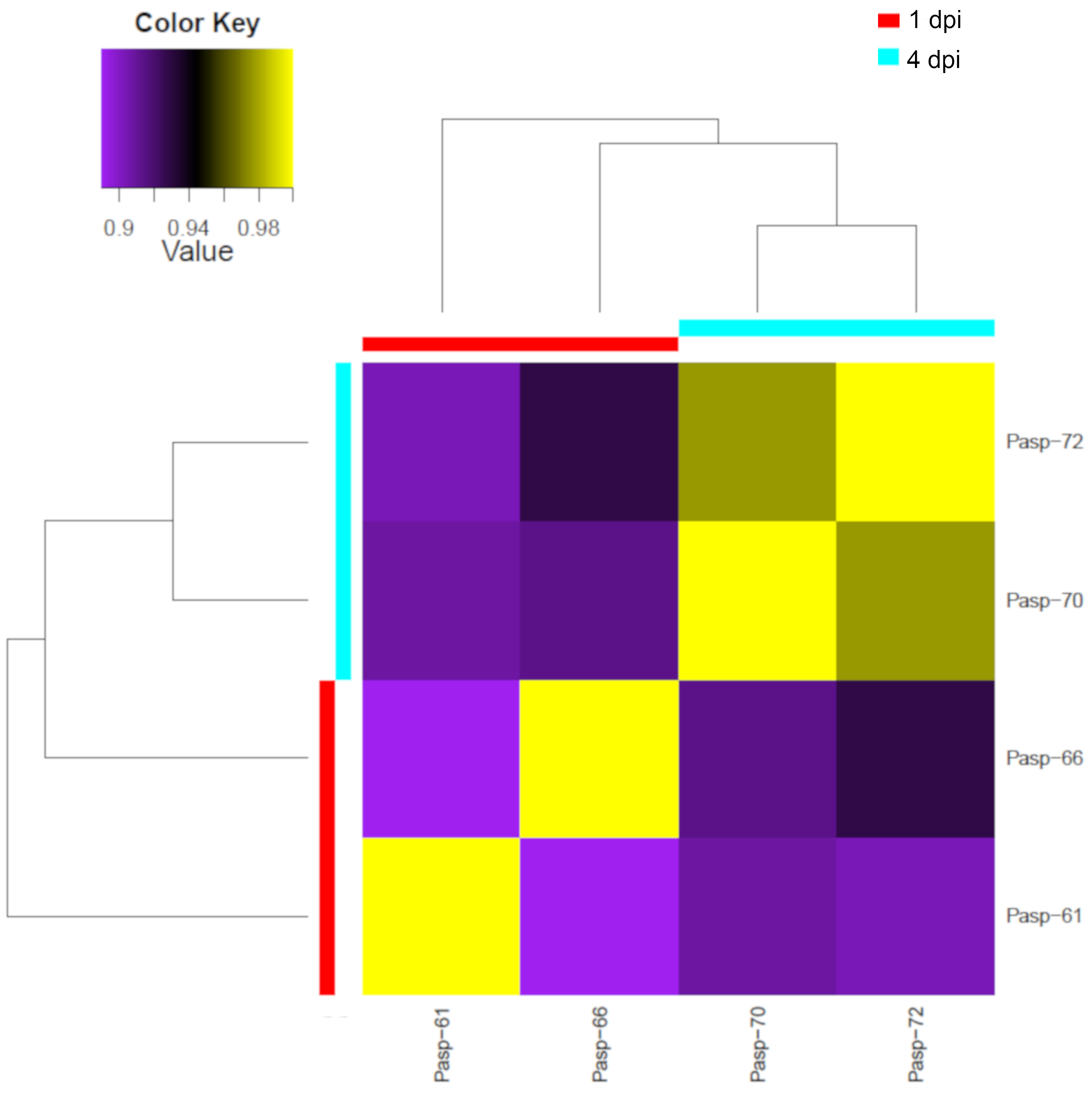

Supplement: Supplementary file 10 — Additional file S10. RNA-seq sample correlation analysis. Clustering analysis of biological replicates between conditions. [file 12864_2021_8077_MOESM10_ESM.tiff]

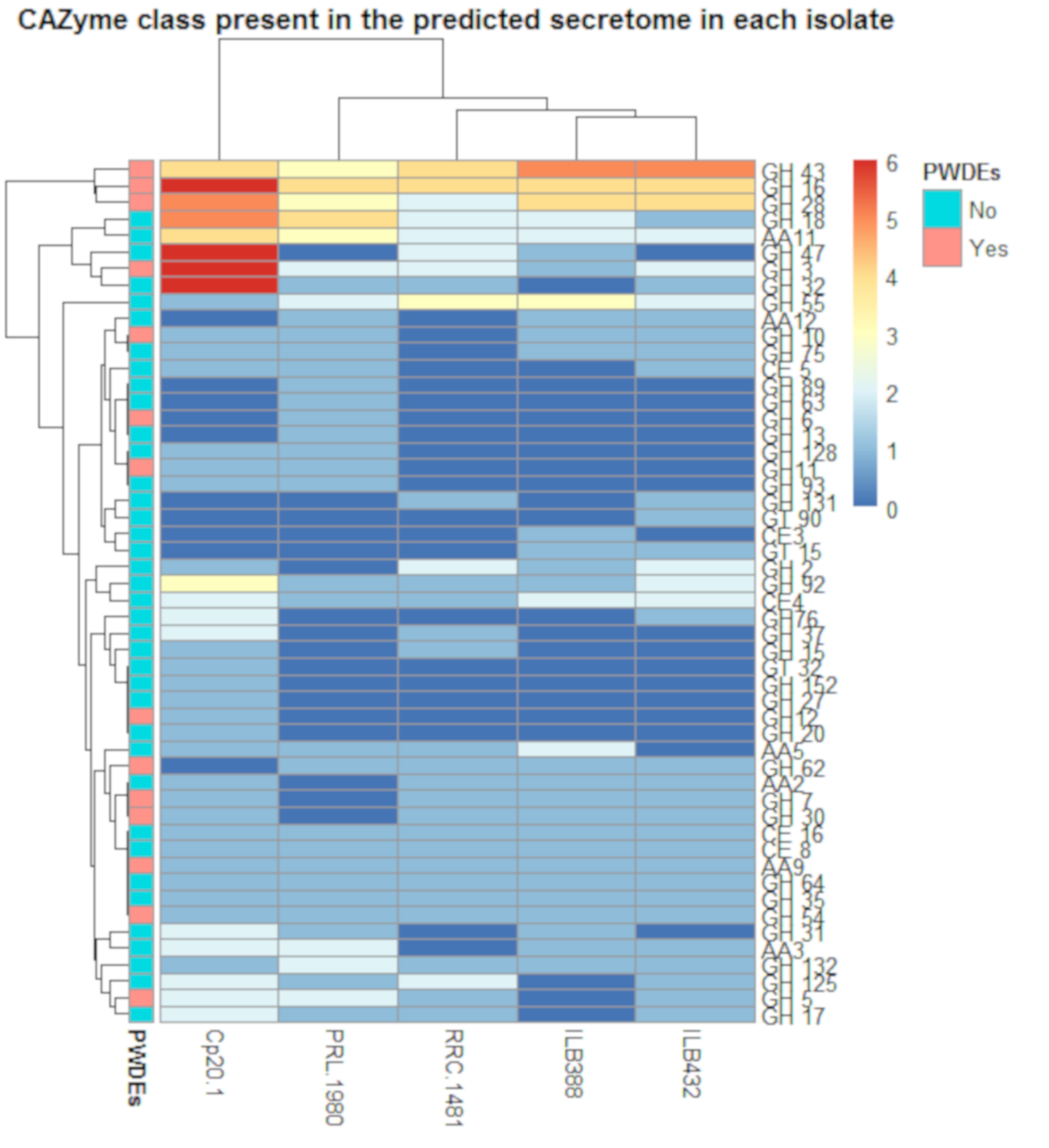

Supplement: Supplementary file 11 — Additional file S11. Representation of CAZymes in each isolate of Claviceps analyzed in this work. Hierarchical clustering and heatmap of CAZymes families in each isolate of Claviceps. [file 12864_2021_8077_MOESM11_ESM.tiff]
